# Cardiotocography and Clinical Risk Factors in Early Term Labor: A Retrospective Cohort Study Using Computerized Analysis With Oxford System

**DOI:** 10.3389/fped.2022.784439

**Published:** 2022-03-16

**Authors:** Aimée A. K. Lovers, Austin Ugwumadu, Antoniya Georgieva

**Affiliations:** ^1^Nuffield Department of Women's and Reproductive Health, Big Data Institute, University of Oxford, Oxford, United Kingdom; ^2^Department of Obstetrics and Gynaecology, St George's, University of London, London, United Kingdom

**Keywords:** cardiotocography, CTG, electronic fetal monitoring, hypoxic-ischaemic encephalopathy, HIE, big data

## Abstract

**Objective:**

The role of cardiotocography (CTG) in fetal risk assessment around the beginning of term labor is controversial. We used routinely collected clinical data in a large tertiary hospital to investigate whether infants with “severe compromise” at birth exhibited fetal heart rate abnormalities in their first-hour CTGs and/or other clinical risks, recorded as per routine care.

**Materials and Methods:**

Retrospective data from 27,927 parturitions (single UK tertiary site, 2001–2010) were analyzed. Cases were included if the pregnancy was singleton, ≥36 weeks' gestation, cephalic presentation, and if they had routine intrapartum CTG as per clinical care. Cases with congenital abnormalities, planned cesarean section (CS), or CS for reasons other than “presumed fetal compromise” were excluded. We analyzed first-hour intrapartum CTG recordings, using intrapartum Oxford System (OxSys) computer-based algorithms. To reflect the effect of routine clinical care, the data was stratified into three exclusive groups: infants delivered by CS for “presumed fetal compromise” within 2 h of starting the CTG (*Emergency CS, n* = 113); between 2 and 5 h of starting the CTG (*Urgent CS, n* = 203); and the rest of deliveries (*Others, n* = 27,611). First-hour CTG and clinical characteristics were compared between the groups, sub-divided to those with and without severe compromise: a composite outcome of stillbirth, neonatal death, neonatal seizures, encephalopathy, resuscitation followed by ≥48 h in neonatal intensive care unit. Two-sample *t*-test, X^2^ test, and Fisher's exact test were used for analysis.

**Results:**

Compared to babies without severe compromise, those with compromise had significantly higher proportion of cases with baseline fetal heart rate ≥150 bpm; non-reactive trace; reduced long-term and short-term variability; decelerative capacity; and no accelerations in the first-hour CTG across all groups. Prolonged decelerations(≥3 min) were also more common. Thick meconium and small for gestational age were consistently more common in compromised infants across all groups. There was more often thick meconium, maternal fever ≥38 C, sentinel events, and other clinical risk factors in the *Emergency CS* and *Urgent CS* compared to the *Others* group.

**Conclusion:**

A proportion of infants born with severe compromise had significantly different first-hour CTG features and clinical risk factors.

## Introduction

Intrapartum hypoxic ischaemic injury is a major contributor to stillbirths, neonatal encephalopathy and mortality, and long-term neurodevelopmental sequelae worldwide. Its prevention is an important public health priority (1–3). Over half of term stillbirths and brain injuries around birth are potentially avoidable by better obstetric care (3–6). Efforts to improve the identification of fetuses at-risk of intrapartum compromise are undermined by the imprecision of currently available tools including the conventional and non-computerized cardiotocograph (CTG) (6–8). The CTG is widely used for assessment of fetal wellbeing and has been the mainstay of fetal monitoring for over 50 years (9, 10). However, CTG interpretation is subjective and relies on the use of guidelines derived from imprecise and static patterns of the fetal heart rate (FHR). Unsurprisingly, delays in intervention and avoidable harm may occur, leading to litigation (10–14). The CTG is also associated with increased operative delivery and healthcare costs (11–13). Therefore, there is increasing interest in more objective and reliable methods for CTG interpretation. This includes computer-based analysis based on a very large routinely collected dataset of CTG and maternity data, such as the Oxford System (OxSys) that is currently under development (15). Moreover, automated CTG evaluation with OxSys enables the analysis of large routinely collected sets of CTG traces, providing evidence for the association of different CTG characteristics, labor management, and labor outcomes, which was the focus of this work.

In current practice, the risk of adverse labor outcomes is based on the assessment of antenatal factors such as abnormal fetal growth, antepartum hemorrhage, prolonged rupture of fetal membranes, meconium staining of the amniotic fluid, or abnormal CTG pattern in the initial assessment of women around labor onset (16, 17). However, the pathophysiology of fetal compromise is complex, multifactorial, and dynamic, and the fetal ability to adapt to oxygen deprivation is significantly challenged by labor and underlying conditions such as placental insufficiency, maternal diabetes, and intrauterine infection, which can weaken the fetal adaptation to oxygen deprivation (18, 19). Furthermore, the role of the CTG in the initial assessment of women around the onset of labor is controversial, because different endpoints are used. Previous randomized controlled trials (RCTs) investigated whether an “admission test” *predicted* neonatal outcome at delivery in the so-called low-risk pregnancy. The primary outcome varied in these different RCTs, including cord acidosis at birth, need for continuous CTG, overall perinatal mortality and morbidity, and operative delivery rates (17, 20, 21). Nonetheless, the original “admission test” aimed to identify the fetus with pre-existing compromise in order to institute obstetric intervention (22). Parts et al. (20) subsequently investigated this original aim in a large retrospective observational study of routine admission CTG in both low- and high-risk pregnancies. The authors investigated “all women who underwent emergency cesarean section due to suspected fetal distress” within 1 h of admission.

Following the objective of the original “admission test” and the selected endpoints by Parts et al., we consider that a more appropriate endpoint for studies of the role of CTG assessment at admission is the early identification of fetuses who are already compromised or vulnerable (e.g., fetal growth restriction, infection, and feto-maternal hemorrhage) and who would benefit from early cesarean delivery in order to avoid further compromise during labor. Therefore, this study aims to evaluate the association between severely compromised infants at birth and early warning signs in their first-hour CTG (recorded before or in first-stage labor as per clinical care) and/or clinical risks, stratifying for those with emergency cesarean section performed shortly after admission due to presumed fetal compromise. We used computerized and classic CTG features, using the OxSys computer-based algorithms, to study this large cohort of infants.

## Materials and Methods

This was a retrospective cohort study of infants delivered at the John Radcliffe Hospital in Oxford, UK, using a clinical data collection system between January 2001 and December 2010. The study received ethical approval from the Newcastle & North Tyneside 1 Research Ethics Committee, Reference 11/NE0044 (data before 2008), and from the South Central Ethics Committee, Reference 13/SC/0153 (for data beyond 2008). Informed consent by the participants was not required.

### Data

**Figure 1:**
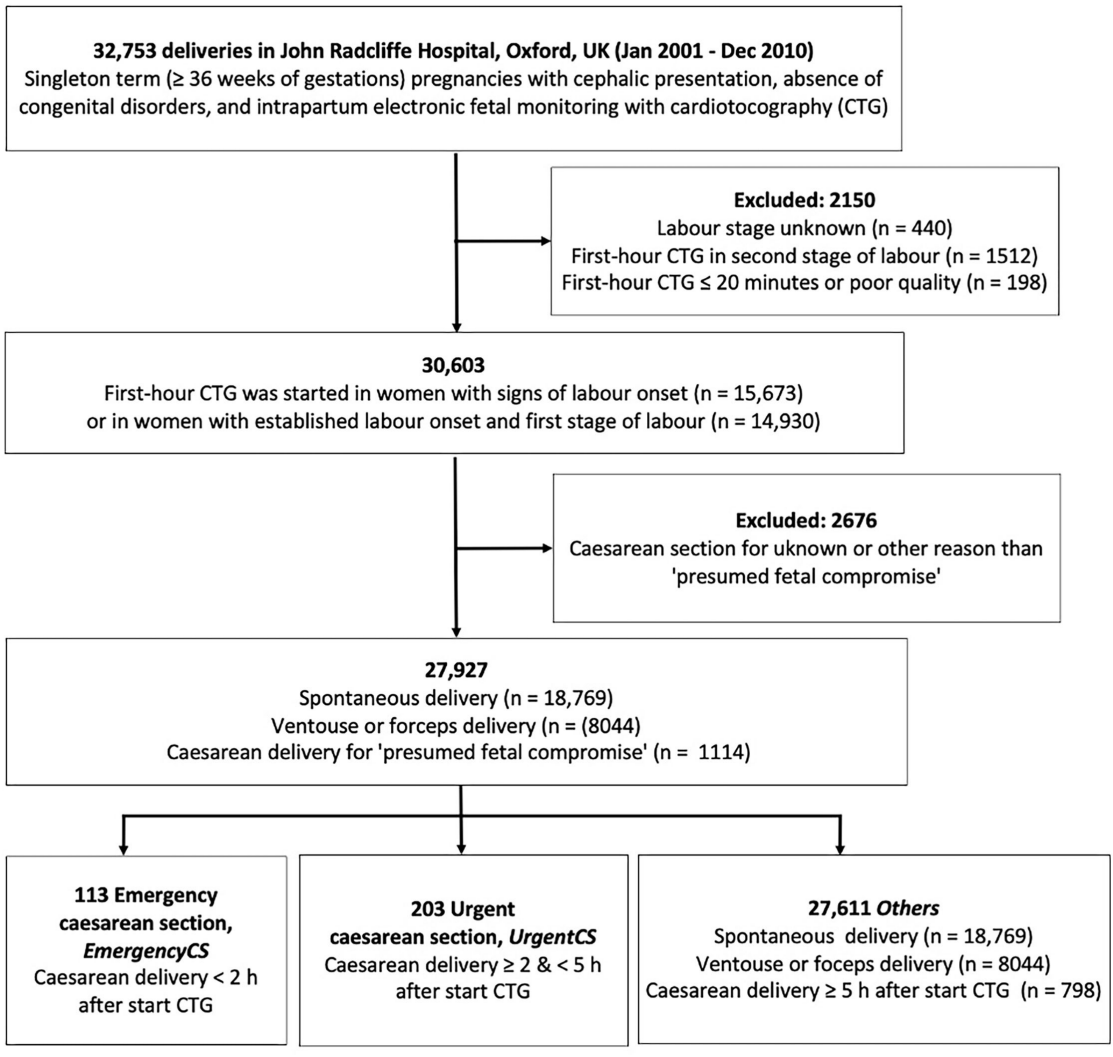
Data flowchart.

During the 10-year study period, there were 32,743 singleton deliveries (excluding elective cesarean sections) with gestational age ≥ 36 weeks of cephalic presentation and normally formed fetuses with intrapartum CTG recorded as per standard care at the John Radcliffe Hospital, Oxford (Figure 1). The selected gestational age of 36 completed weeks is in line with the threshold used in our previous work and in large randomized controlled trials on intrapartum CTG (23–25). We only included cases with at least 20 min of CTG recordings of acceptable signal quality for analysis, taken only at maternity admission or delivery units. The national guidance at the time and still is intermittent auscultation for the intrapartum surveillance of low-risk pregnancies, defined as the absence of any antenatal or intrapartum, fetal, maternal, placental, medical, or obstetric complications or risk factors. Figure 1 shows the study data flow chart. We excluded cases where the CTG recording commenced in the second stage of labor and those with cesarean delivery for indication other than “presumed fetal compromise.” A total of 27,927 deliveries were eligible for inclusion and analysis.

### Methods

All data were routinely collected as part of standard clinical care and analyzed retrospectively. Outcome groups were based on the mode and timing of cesarean delivery for presumed fetal compromise (Figure 1). Infants delivered by cesarean section within 2 h of CTG monitoring (emergency cesarean delivery, *Emergency CS*) were compared with their counterparts delivered by cesarean section between 2 and 5 h (urgent cesarean delivery, *Urgent CS*). The remaining births formed the control group (*Others*).

*Initial assessment* was defined as the assessment of fetal wellbeing and clinical risk factors in women presenting to the unit with symptoms and signs of labor.

Sentinel events included placental abruption, uterine rupture, and cord prolapse. Birth trauma included intracranial laceration and hemorrhage due to birth injury; other birth injuries to central nervous system; birth injury to skeleton; and birth injury to peripheral nervous system.

Birth weight deviations were calculated with the adjusted Yudkin's chart percentiles (26) and reported in terms of small for gestational age (birth weights below the 3rd percentile) and large for gestational age (birth weights above the 97th percentile).

*Severe compromise* was defined as a composite outcome consisting of clinically relevant short-term perinatal outcomes collected at hospital level including stillbirth, neonatal death, seizures, neonatal encephalopathy, and need for intubation or intensive resuscitation followed by neonatal intensive care unit (NICU) admission for ≥48 h (15). Reported birth outcomes include *severe compromise*, Apgar score <4 at 1 min, Apgar score <7 at 5 min, and/or umbilical cord arterial pH <7.0 or <7.05 (27).

*Onset of labor* was defined by the attending clinician as per clinical practice (regular uterine contractions associated with cervical dilatation ≥3 cm) and documented in the routinely collected digital maternity notes.

Digital CTG data, sampled at 4 Hz as per the standard output of fetal monitors (typically Philips Avalon) were archived by a central monitoring system (Huntleigh Healthcare Ltd., Cardiff, UK). Computerized CTG features were calculated with the Oxford System (OxSys1.5) algorithms (15). OxSys analyses the CTG recordings in 15 min windows that move forward every 5 min; CTG characteristics are calculated for each window after heuristic noise removal algorithms. Pregnancy and labor data were derived from the Oxford Clinical Maternity Database, detailing maternal demographics, obstetric history, labor, delivery outcomes, and infant characteristics.

We considered only the first available 20–60 min of the CTG traces as part of the *initial assessment* (28), based on the recommended 20 min duration for CTG around labor onset (22) and additional time for the assessment of fetal behavioral states if they were absent in the first 20 min. Where available, the entire first hour of CTG was analyzed and the results reported. Where not, the longest available CTG segment (≥20 min) was analyzed instead. CTG recordings made after the first hour were not analyzed in this study.

In addition to standard CTG characteristics such as baseline rate, variability, decelerations, and accelerations, OxSys1.5 computes the decelerative capacity (DC), analyzing the entire FHR signal in a moving 15 min window to provide an average measure of downward movements (29). Lower DC values are measured in a normal trace without decelerations, whereas increased DC values are measured in deep, steep-sloped, and/or repetitive decelerations (15). Non-reactive trace is defined as DC <1 bpm and no accelerations as previously reported (15).

### Statistical Methods

Data were analyzed in MATLAB version R2019b (The MathWorks Inc., Massachusetts, USA). Medians with 25 and 75th percentiles were calculated for continuous and ordinal variables. Incidence with percentages were calculated for categorical variables. Univariate analysis of categorical variables was performed with the X^2^ test and Fisher's exact test with relative risks, odds ratios, and 95% confidence intervals as appropriate. The Kruskal-Wallis one-way analysis of variance test was used to compare the medians and interquartile range (IQR) of continuous variables.

## Results

The CTG recording was commenced at different times in relation to the clinically determined time of the onset of active labor. As a result, 90% of all traces were within 10 h before and 6.5 h after labor onset (second stage recordings were excluded). The median difference between labor onset and CTG start was 7 min. The interquartile range is−135 and 230 min, negative values correspond to CTGs started after labor onset (46% of all), and positive values to those started before labor onset (56%).

Cesarean delivery for presumed fetal compromise was performed within 2 h of starting the CTG (*Emergency CS*) in 0.4% (113/27,927) and between 2 and 5 h (*Urgent CS*) in 0.7% (203/27,927) of all deliveries.

**Figure 2:**
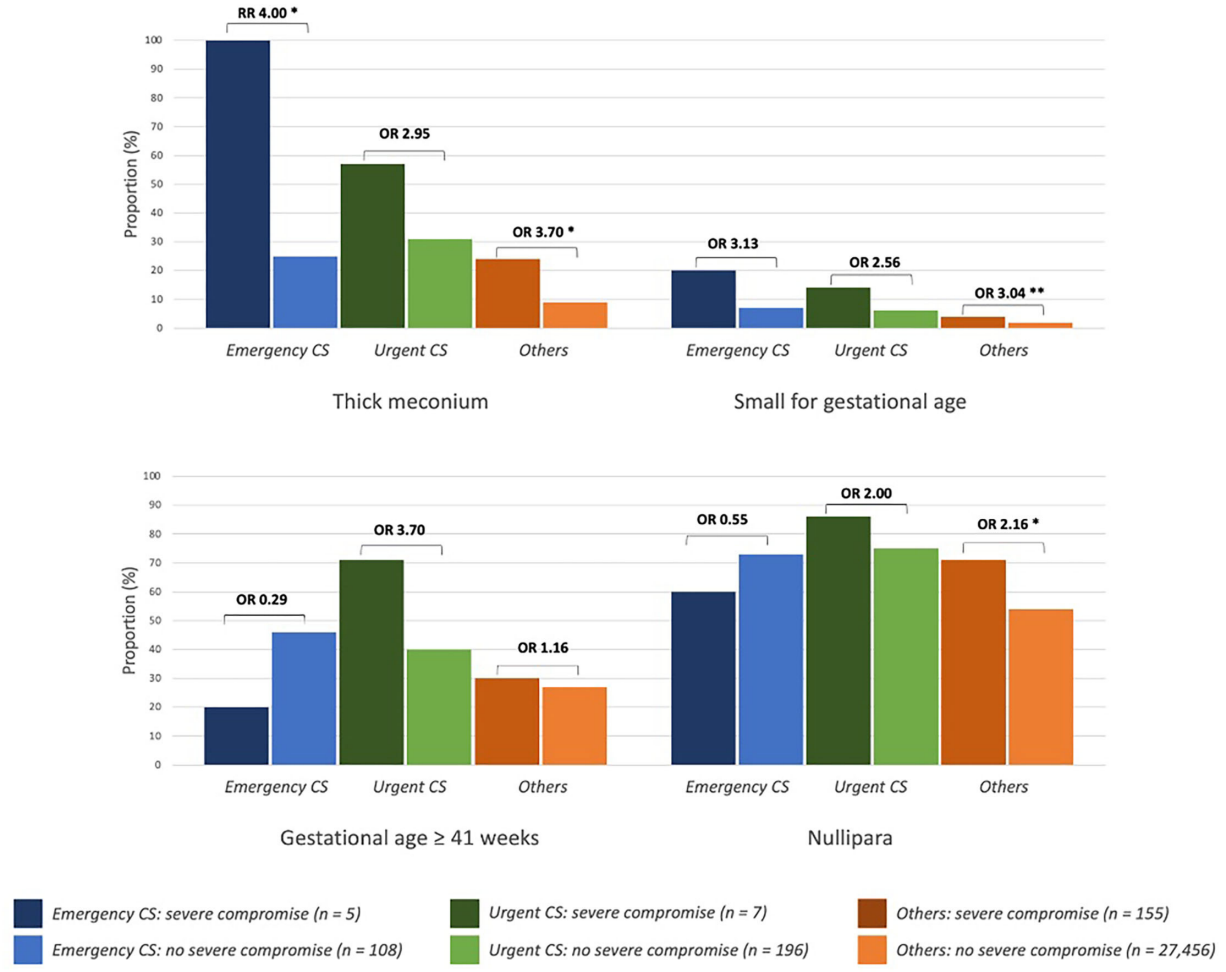
Clinical characteristics of newborns with and without severe compromise. *Emergency CS*, emergency caesarean delivery (<2 h after start CTG monitoring); *Urgent CS*, urgent caesarean delivery (between 2 to 5 h after start CTG monitoring); RR, risk ratio; OR, odds ratio; *p ≤ 0.01, **p ≤ 0.05.

**Figure 3:**
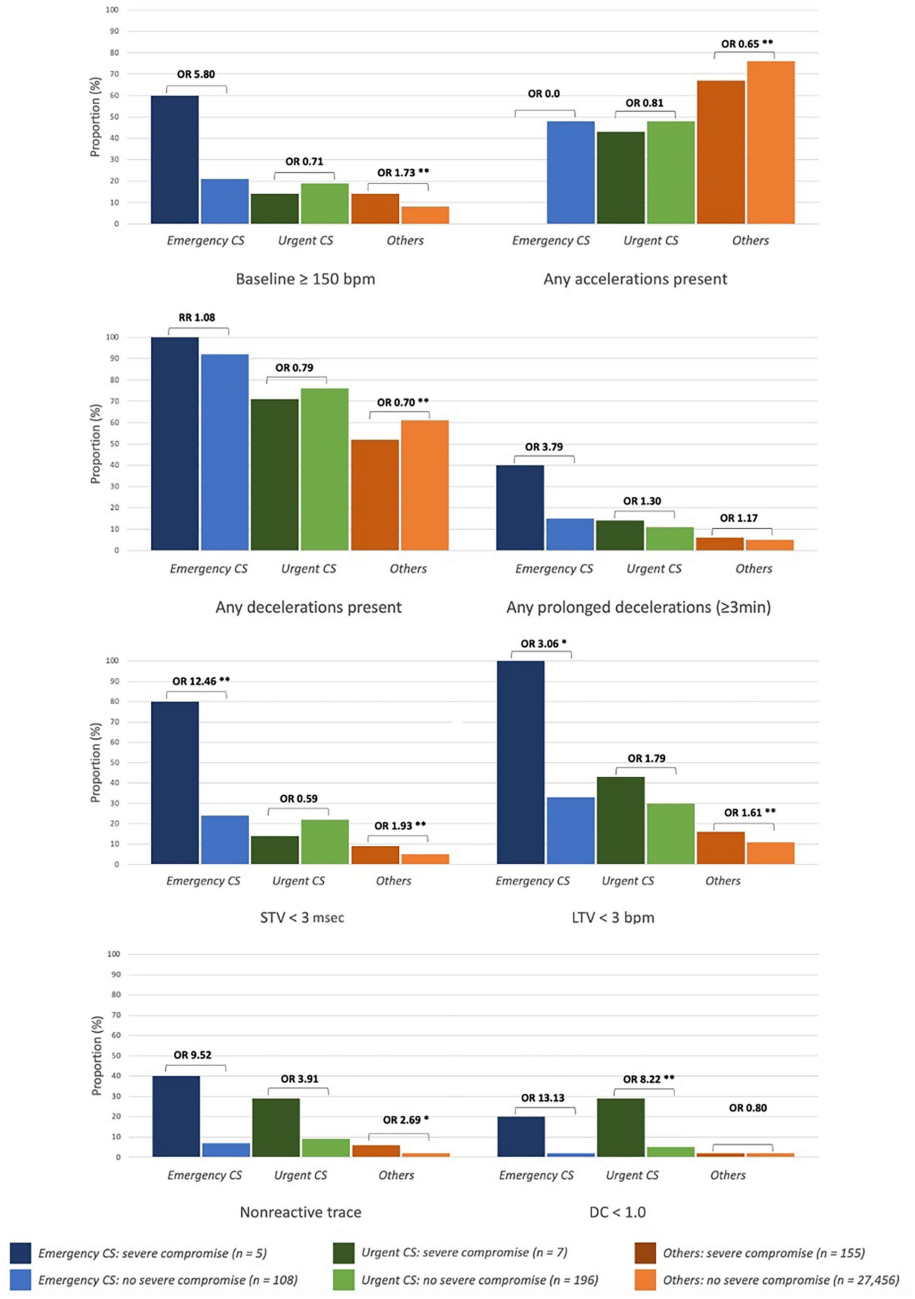
First-hour CTG characteristics of newborns with and without severe compromise. *Emergency CS*, emergency caesarean delivery (<2 h after start CTG monitoring); *Urgent CS*, urgent caesarean delivery (between 2 to 5 h after start CTG monitoring); DC, decelerative capacity; LTV, long-term variability; RR, risk ratio; OR, odds ratio. *p ≤ 0.01, **p ≤ 0.05.

Majority of infants with severe compromise at birth were in *Others* (*n* = 155 at rate of 0.6%), with another five and seven in the *Emergency CS* and *Urgent CS* groups respectively (rates of 4.4 and 3.4%, respectively). Figures 2, 3 show the proportion of babies with selected characteristics of interest for the three groups (*Emergency CS, Urgent CS*, and *Others*), stratified for neonatal outcome. Dark colors correspond to the proportion of severely compromised babies and light colors to those without severe compromise.

Thick meconium and small for gestational age were more common in deliveries with severely compromised neonates across all groups; nulliparous women were more common in *Others* with severe compromise vs. *Others* without severe compromise (Figure 2). Post-date deliveries, induced labor, and maternal fever were more common clinical characteristics of severely compromised babies in the *Urgent CS* group and *Others*.

Figure 3 shows the first-hour CTG characteristics of severely compromised babies compared to those without severe compromise. Baseline ≥150 bpm, non-reactive trace, reduced long-term variability (LTV), and fewer accelerations were more prevalent in severely compromised neonates in all groups. Baseline FHR ≥150 bpm was more common in severely compromised babies in the *Emergency CS* group and *Others*. Reduced DC <1 bpm was significantly more common in severely compromised newborns with *Urgent CS*.

Selected details of clinical and CTG characteristics are shown in Table 1 and the full details in the Supplementary Table 1.

**Table 1:** Clinical and CTG characteristics of total cohort (*n* = 29,927).

	**Emergency cesarean section, *Emergency CS* (*n* = 113)**		**Urgent cesarean section, *Urgent CS* (*n* = 203)**		**All *Others* (*n* = 27,611)**		
	***n* or *median***	**% or *IQR***	***n* or *median***	**% or *IQR***	***n* or *median***	**% or *IQR***	***p*-value**
**LABOR DETAILS**							
**Labor onset**							
Established labor at start CTG	103	91.2	135	66.5	13,485	48.8	≤ 0.01
**Antepartum risk factors**							
Nulliparity	82	72.6	153	75.4	14,901	54.0	≤ 0.01
Post term (≥41 weeks)	51	45.1	84	41.4	7,522	27.2	≤ 0.01
**Intrapartum risk factors**							
Maternal fever (≥38.0°C)^†^	12^A^	12.4^A^	20^B^	11.2^B^	1,482^C^	5.6^C^	≤ 0.01
Thick meconium	32	28.3	65	32.0	2,182	7.9	≤ 0.01
Sentinel event	6	5.3	8	3.9	130	0.5	≤ 0.01
**Delivery mode**							
Cesarean section	113	100.0	203	100.0	798	2.9	–
Instrumental vaginal	–	–	–	–	8,044	29.1	–
Spontaneous vaginal	–	–	–	–	18,769	68.0	–
**DELIVERY OUTCOME**
**Objective fetal compromise**	
Severe compromise*^‡^*	5	4.4	7	3.4	155	0.6	≤ 0.01
Resuscitation	4	3.5	9	4.4	199	0.7	≤ 0.01
Apgar score <4 at 1 min	9	8.0	24	11.8	591^D^	2.1^D^	≤ 0.01
Apgar score <7 at 5 min	3	2.7	8	3.9	229^E^	0.8^E^	≤ 0.01
Arterial umbilical cord pH	7.23^F^	7.15−−7.28^F^	*7.22* ^F^	7.12−−7.27^F^	7.22^G^	7.14−−7.28^G^	≤ 0.01
pH <7.00	6^F^	2.8^F^	9^F^	4.6^F^	190^G^	1.0^G^	≤ 0.01
pH <7.05	9^F^	8.4^F^	23^F^	11.7^F^	502^G^	2.5^G^	≤ 0.01
**Mortality**							
Stillbirth	0	0.0	0	0.0	0	0.0	–
Neonatal death	1	0.9	0	0.0	16	0.1	≤ 0.01
**Morbidity**							
Convulsions	3	2.7	1	0.5	43	0.2	≤ 0.01
Meconium aspiration syndrome	9	8.0	7	3.4	78	0.3	≤ 0.01
NICU admission	12	10.6	32	15.8	1,133	4.1^E^	≤ 0.01
Length of stay (days)	*6*	*2–10*	*4*	*2–7*	3	*1–5*	0.109
**COMPUTERIZED CTG FEATURES (FIRST HOUR)**							
Baseline (bpm)	138^H^	129−−148^H^	139^I^	130−−148^I^	135^J^	127−−142^J^	≤ 0.01
≥150 bpm	25^H^	22.1^H^	38^I^	18.9^I^	2,299^J^	8.4^J^	≤ 0.01
≥160 bpm	14^H^	12.5^H^	11^I^	5.5^I^	585^J^	2.1^J^	≤ 0.01
Short-term variability	5.4^H^	2.9−−9.4^H^	4.6^I^	3.1−−6.9^I^	6.1^J^	4.5−−7.9^J^	≤ 0.01
<3 msec	30^H^	26.8^H^	44^I^	21.9^I^	1,358^J^	5.0^J^	≤ 0.01
Long-term variability	3.9^H^	2.5−−6.3^H^	3.7^K^	2.8−−4.9^K^	4.9^L^	3.8−−6.2^L^	≤ 0.01
<3 bpm	40^H^	35.7^H^	60^K^	30.0^K^	2,961^L^	10.9^L^	≤ 0.01
Non-reactive trace	9^H^	8.0^H^	20^I^	10.0^I^	624^M^	2.3^M^	≤ 0.01
Any accelerations present	51^H^	45.5^H^	96^I^	47.8^I^	20,572^M^	75.5^M^	≤ 0.01
Any decelerations present	103^I^	92.8^I^	150^N^	75.8^N^	16,2431^O^	61.29^O^	≤ 0.01
Any prolonged decelerations (≥3 min)	18^H^	16.0^H^	23^I^	11.4^I^	1,388^M^	5.1^M^	≤ 0.01
Decelerative capacity (bpm)	3.2^H^	2.0−−4.6^H^	2.3^I^	1.6−−3.2^I^	2.5^J^	1.9−−3.3^J^	≤ 0.01
<1.0 bpm	3^H^	2.7^H^	11^I^	5.5^I^	664^J^	2.5^J^	≤ 0.01

*n, number; IQR, inter-quartile range (25–75th percentiles); NICU, neonatal intensive care unit; bpm, beats per minute. Super-indices A–O indicate the number of missing data: A, 16; B, 24; C, 1074; D, 8; E, 11; F, 6; G, 7811; H, 1; I, 2; J, 362; K, 3; L, 388; M, 359; N, 3; O, 752.*

† *Maternal fever defined as one-time maximum maternal temperature measurement of ≥38.0°C, based on UK maternity guidelines (29).*

‡ *Composite outcome: stillbirth, neonatal death, seizures, neonatal encephalopathy, intubation or resuscitation followed by NICU admission for ≥ 48 h*.

Table 2 details clinical and CTG characteristics specifically for the neonates with severe compromise at birth (*n* = 167), and full details are in the Supplementary Table 2. These represent 4.4% (5/113) of babies with *Emergency CS*; 3.4% (7/203) of babies with *Urgent CS*; and 0.6% (155/27,611) of *Others*.

**Table 2:** Clinical and CTG characteristics of severely compromised newborns (*n* = 167).

	**Emergency cesarean section, *Emergency CS* (*n* = 5)**		**Urgent cesarean section, *Urgent CS* (*n* = 7)**		**All *Others* (*n* = 155)**		
	***n* or *median***	**% or *IQR***	***n* or *median***	**% or *IQR***	***n* or *median***	**% or *IQR***	***p*-value**
**LABOR DETAILS**
**Labor onset**
Established labor at start CTG	5	100.0	5	71.4	54	34.8	≤ 0.01
**Antepartum risk factors**
Nulliparity	3	60.0	6	85.7	111	71.6	0.601
Post term (≥41 weeks)	1	20.0	5	71.4	47	30.2	0.062
**Intrapartum risk factors**
Maternal fever (≥38.0°C)^†^	0	0.0	1^A^	16.7^A^	13^B^	8.6^B^	0.618
Thick meconium	5	100.0	4	57.1	37	23.9	≤ 0.01
Sentinel event	0	0.0	0	0.0	1	0.6	0.962
**Delivery mode**							
Cesarean section	5	100.0	7	100.0	19	12.3	–
Instrumental vaginal	–	–	–	–	62	40.0	–
Spontaneous vaginal	–	–	–	–	74	47.7	–
**DELIVERY OUTCOME**
**Objective fetal compromise**
Severe compromise*^‡^*	5	100.0	7	100.0	155	100.0	–
Resuscitation	3	60.0	6	85.7	98	63.2	0.470
Apgar score <4 at 1 min	1	20.0	6	85.7	85^A^	55.2^A^	0.076
Apgar score <7 at 5 min	1	20.0	4	57.1	75 ^C^	49.0^C^	0.396
Arterial umbilical cord pH	*7.14*	*7.09–7.28*	6.83^A^	6.78−−7.01^A^	7.14^D^	7.05−−7.23^D^	≤ 0.01
pH <7.00	0	0.0	4^A^	66.7^A^	26^D^	19.3^D^	≤ 0.01
pH <7.05	1	20.0	5^A^	88.3^A^	33^D^	24.4^D^	≤ 0.01
**Mortality**							
Stillbirth	0	0.0	0	0.0	0	0.0	–
Neonatal death	1	20.0	0	0.0	14	9.0	0.488
**Morbidity**							
Convulsions	3	60.0	1	14.3	43	27.7	0.203
Meconium aspiration syndrome	5	100.0	1	14.3	19	12.3	≤ 0.01
NICU admission	5	100.0	6	85.7	131	84.5	0.633
Length of stay (days)	*9*	*8–18*	*5*	*4–6*	***3***	*3–8*	0.168
**COMPUTERIZED CTG FEATURES (FIRST HOUR)**							
Baseline (bpm)	*158*	*145–164*	*135*	*127–145*	136^C^	129−−144^C^	0.014
≥150 bpm	3	60.0	1	14.3	21^C^	13.7^C^	0.027
≥160 bpm	2	40.0	0	0.0	8^C^	5.2^C^	≤ 0.01
Short-term variability	*1.8*	*1.6–2.7*	5.6	*3.2–7.0*	5.3^C^	4.1−−7.8^C^	≤ 0.01
<3 msec	4	80.0	1	14.3	14^C^	9.2^C^	≤ 0.01
Long-term variability	*2.1*	*1.6–2.6*	*4.4*	*2.1–5.2*	4.6^C^	3.4−−6.1^C^	≤ 0.01
<3 bpm	5	100.0	3	42.9	25^C^	16.3^C^	≤ 0.01
Non-reactive trace	2	40.0	2	28.6	9^C^	5.9^C^	≤ 0.01
Any accelerations present	0	0.0	3	42.9	102^C^	66.7^C^	0.013
Any decelerations present	5	100.0	5	71.4	80^A^	52.6^A^	0.125
Any prolonged decelerations (≥3 min)	2	40.0	1	14.3	9 ^C^	5.8^C^	0.012
Decelerative capacity (bpm)	*1.8*	*1.0–2.4*	*2.3*	*1.2–3.8*	2.4^C^	1.7−−3.1^C^	0.301
<1.0 bpm	1	20.0	2	28.6	3^C^	2.0^C^	≤ 0.01

*n, number; IQR, inter-quartile range (25–75th percentiles); NICU, neonatal intensive care unit; bpm, beats per minute. Super-indices A–D indicate the number of missing data: A, 1; B, 4; C, 2; D, 20.*

† *Maternal fever defined as one-time maximum maternal temperature measurement of ≥38.0°C, based on UK maternity guidelines (29).*

‡ *Composite outcome: stillbirth, neonatal death, seizures, neonatal encephalopathy, intubation or resuscitation followed by NICU admission for ≥ 48 h*.

**Figure 4:**
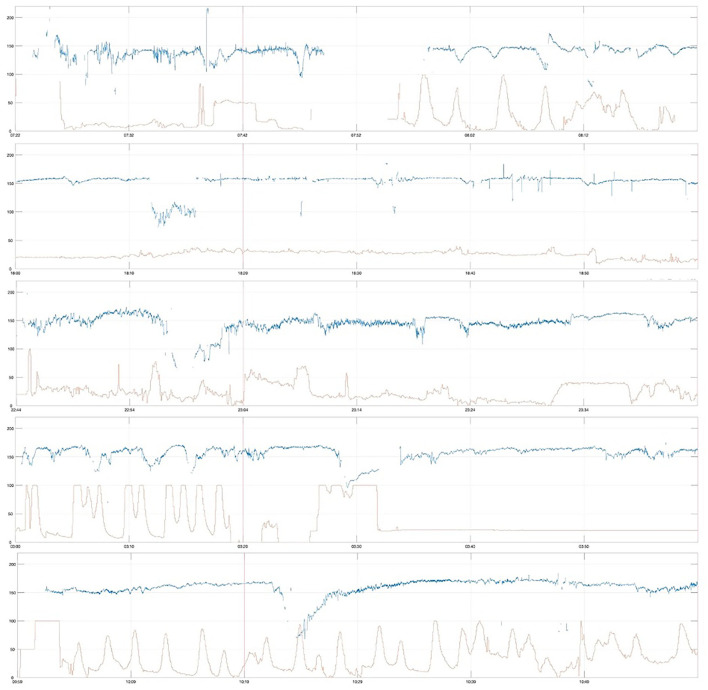
First-hour CTG of severely compromised newborns delivered by cesarean section within 2 h after CTG start (emergency cesarean delivery, *Emergency CS*), vertical red line at 20 min, paper speed 1 cm/min, vertical scale 20 beats per min/cm.

All severely compromised neonates in the *Emergency CS* group (*n* = 5) had thick meconium, meconium aspiration syndrome, and were admitted to the neonatal intensive care unit (NICU), and all of these were less common in the severely compromised infants in the other two groups. Their first-hour CTG traces were marked by baseline ≥150 and ≥160 bpm; reduced variability (both short-term variability (STV) and LTV); non-reactive trace; prolonged decelerations; and lack of accelerations. The first-hour CTGs are shown in Figure 4.

**Figure 5:**
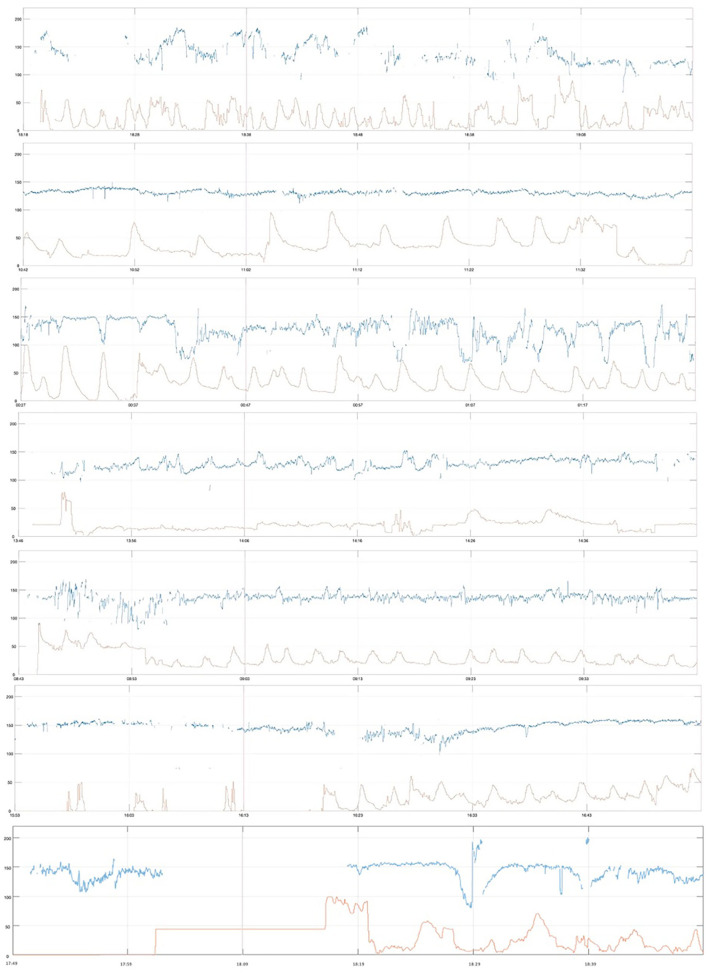
First-hour CTG of severely compromised newborns delivered by cesarean section between 2 and 5 h after start of CTG (urgent cesarean delivery, *Urgent CS*), vertical red line at 20 min, paper speed 1 cm/min, vertical scale 20 beats per min/cm.

Severely compromised babies in the *Urgent CS* group (*n* = 7) were more often induced post-date labors in first-time mothers (Table 2) and had depressed Apgar scores and critically low umbilical cord arterial pH (group median pH was 6.83). Their first-hour CTG traces were marked by reduced variability (particularly LTV) and non-reactive trace, and the CTGs are shown in Figure 5.

**Figure 6:**
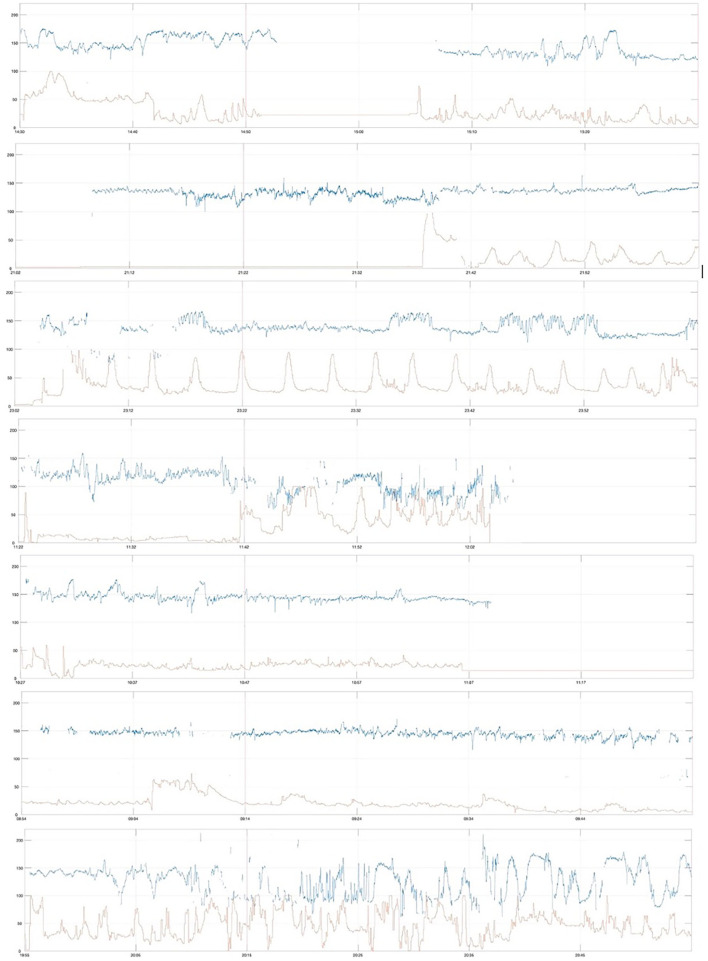
First-hour CTG traces of severely compromised newborns of the remaining cohort (*Others*), random selection of 8 traces, vertical red line at 20 min, paper speed 1 cm/min, vertical scale 20 beats per min/cm.

Severely compromised neonates in *Others* (*n* = 155) were delivered by spontaneous vaginal delivery in 47.7% (74/155), instrumental vaginal delivery in 40% (62/155), and by cesarean section in 12.3% (19/155). Their first-hour CTG traces were marked by reduced variability (both LTV and STV) and non-reactive traces. A random selection of eight of these first-hour CTGs is shown in Figure 6.

## Discussion

### Main Findings

Analysis of this routinely collected cohort of over 27,000 term births showed that an important proportion of fetuses with severe compromise or at risk of severe compromise had significantly different features of the first-hour CTG and presence of clinical risk factors such as thick meconium, small for gestational age, and maternal fever ≥38C. We considered three exclusive groups: infants delivered by CS for “presumed fetal compromise” within 2 h of starting the CTG (*Emergency CS*); between 2 and 5 h of starting the CTG (*Urgent CS*); and the rest of deliveries (*Others*). This data stratification allowed us to account for any effects the emergency or urgent cesarean sections had on labor outcomes, as well as to obtain estimates for the proportion of severely compromised infants for whom potential early signs were present. Severely compromised infants in these groups had higher rates of baseline FHR ≥150 bpm, non-reactive initial CTG, reduced short-term and long-term variability, less accelerations, and decelerative capacity amongst fetuses with severe compromise. Prolonged decelerations (≥3 min) were also more common in the first hour CTG of infants born with severe compromise. None of the severely compromised infants in the *Urgent CS* had a first hour baseline ≥160 bpm and only a small proportion had STV <3 msec. In this group, non-reactive initial trace and DC <1 bpm were stronger risk factors in about a third of severely compromised infants. There was a significantly higher proportion of babies with arterial cord pH <7.00 in the severely compromised babies with *Urgent CS* (Table 2), with the length of stay in NICU longest for the severe compromised babies in the *Emergency CS* group, suggesting that the components of the “severe compromise” varied between the groups. Umbilical artery acidemia was absent in the majority of severely compromised infants in *Emegency CS* and *Others*. The rate of decelerations in the initial CTG was not associated with severe compromise, but our data was “censored” by clinical practice.

In addition, we demonstrated that computerized analysis of the CTG adds value by permitting the analysis of large cohorts to support clinical guidelines.

### Clinical Implications and Interpretation

Our data suggest that clinical guidelines for CTG interpretation for the *initial* trace may need to differ from those applied to the rest of labor. Perhaps there should be less emphasis on the presence of decelerations *per se*, unless they are prolonged (≥3 min). Instead, there should be a lower threshold for the definition of FHR “tachycardia” at this time (i.e., 150 bpm), an assessment for a non-reactive trace (i.e., lack of cyclicity or reactivity). Our data also confirm the relevance of and provide evidence for STV and LTV, as well as clinical risk factors such as thick meconium, small for gestational age, and others.

These findings suggest that a consistent assessment of the CTG together with known clinical risk factors around the onset of labor has the potential to identify early a small but important proportion of babies at risk of severe compromise. Our data shows that it is likely that some of the cases were “missed” in the group of *Others* and recognized late in the *Urgent CS* group. Some of the CTG characteristics important for the first-hour CTG assessment, such as reduced variability, decelerative capacity, and the non-reactive trace, are not typically detectable with intermittent auscultation (20).

The primary outcome of “severe compromise” was broadly defined to include cases which may not have their origins in hypoxic-ischaemic insults. Approximately half of small for gestational age fetuses attributable to placental insufficiency are missed in the antenatal period, and about 10% of them sustain severe compromise (30). Other vulnerabilities, including intrauterine infection, cerebral hemorrhage, and maternal hyperglycaemia (13, 18, 19, 31), may also alter fetal physiological adaptations to reduced oxygen during labor (18, 19) in a manner that is difficult to detect with conventional CTG (13, 18, 31). Therefore, in principle, clinical factors and fetal physiology should be considered for accurate interpretation of CTG in the initial assessment of women in labor. Our findings confirm the importance of both first-hour CTG and clinical characteristics.

Our findings are relevant for fetuses for whom cesarean delivery before or in early labor may avoid the more dramatic emergency deliveries and the attendant maternal and fetal risks (30). Furthermore, better risk assessments with CTG in the initial assessment could enhance individualized care, informed decision-making, and improve maternal engagement during labor (17, 32).

### Strengths and Limitations

Using computerized methods, we studied a large, detailed, and complete, high-quality maternity database. Specific markers of brain development were not collected at the hospital level and therefore unavailable for our data analysis of routinely collected cohort data. We included all eligible infants resulting in nearly equal proportion of babies with CTG starting before and after the onset of labor, 46 vs. 54% respectively. Therefore, our findings are relevant to both periods, pre and early labor, largely around onset of labor where women are assessed in a maternity unit triage room or bays. This is particularly important because the definition of onset of labor and CTG initiation varies and depends, amongst other things, on the time the woman presents to the hospital.

Clinical practice has inevitably changed since the timespan of this dataset and the data for the subsequent 10-year period (2011–2020) were not fully available yet at the time of analysis, but their analysis will follow the presented format in this study to allow comparison.

A limitation of this study was the lack of information for the cervical dilatation at the time of initial CTG recording as well as the fact that our routinely collected data relate to a high-risk obstetric population, as per clinical CTG use in the UK.

We propose to build on the various univariate analyses presented in the current study to undertake multivariate analyses in future studies using larger datasets.

## Conclusion

This study reports that a small but important proportion of infants born with severe compromise had significantly different computerized CTG characteristics around the onset of labor (detected with OxSys algorithms), typically in the context of other clinical risk factors. Clinical guidelines for CTG interpretation, for the *initial* trace specifically, may need to be different from those for monitoring throughout labor, with less focus on the presence of decelerations *per se*, unless they are prolonged (≥3 min). Instead, there should be a lower threshold for the definition of FHR “tachycardia” and assessment for non-reactive trace (lack of cyclicity or reactivity). We further confirm the relevance of and provide evidence for STV and LTV, as well as clinical risk factors including thick meconium and small for gestational age.

## Data Availability Statement

The data analyzed in this study is subject to the following licenses/restrictions: The Ethics Approval does not allow for sharing the data publicly. Requests to access these datasets should be directed to antoniya.georgieva@wrh.ox.ac.uk.

## Ethics Statement

The studies involving human participants were reviewed and approved by Newcastle & North Tyneside 1 Research Ethics Committee, Reference 11/NE0044 (data before 2008), and from the South Central Ethics Committee, Reference 13/SC/0153 (for data beyond 2008). Written informed consent for participation was not provided by the participants' legal guardians/next of kin because: This was routinely collected and fully anonymised clinical data, and no personal information was processed.

## Author Contributions

AG conceived the study, pre-processed the data, and analyzed the CTGs. AL analyzed the full data, conducted the literature review, and wrote the first manuscript draft. AU provided clinical oversight and input to the research question, data analysis, and manuscript. All authors reviewed and contributed to writing the manuscript.

## Funding

AG was funded by the UK National Institute of Health Research (CDF-2016-09-004). The views expressed here are those of the authors and not necessarily those of the NHS, the NIHR or the Department of Health.

## Author Disclaimer

The views expressed here are those of the authors and not necessarily those of the NIHR or the Department of Health (UK).

## Conflict of Interest

The authors declare that the research was conducted in the absence of any commercial or financial relationships that could be construed as a potential conflict of interest.

## Publisher's Note

All claims expressed in this article are solely those of the authors and do not necessarily represent those of their affiliated organizations, or those of the publisher, the editors and the reviewers. Any product that may be evaluated in this article, or claim that may be made by its manufacturer, is not guaranteed or endorsed by the publisher.
