# Supplementary material for: Cardiotocography and Clinical Risk Factors in Early Term Labor: A Retrospective Cohort Study Using Computerized Analysis With Oxford System
Source: Front Pediatr. 2022 Mar 16;10:784439. doi: 10.3389/fped.2022.784439 (PMC8966702; doi:10.3389/fped.2022.784439)
Supplement: Supplementary file 1 [file Data_Sheet_1.PDF]

Supplementary Material

Role of cardiotocography in early term labour for the detection of fetuses at risk of intrapartum fetal compromise: a retrospective cohort study

Aimée A K Lovers, Austin Ugwumadu, Antoniya Georgieva

Supplementary Table 1. Clinical and CTG characteristics of total cohort (n = 29,927)

	**Emergency caesarean section, *EmergencyCS* (*n* = 113)**		**Urgent caesarean section, *UrgentCS* (*n* = 203)**		**All *Others* (*n* = 27,611)**		
	n or *median*	% or *IQR*	n or *median*	% or *IQR*	n or *median*	% or *IQR*	p-value
**LABOUR DETAILS**							
**Labour onset**							
Induced labour	52	46.0	85	41.9	12,658	45.8	0.527
Established labour at start CTG	103	91.2	135	66.5	13,485	48.8	≤ 0.01
**Intrapartum risk factors**							
Augmentation with oxytocin	9	8.0	59	29.1	10,459	37.9	≤ 0.01
Maternal temperature (°C) *	***36.6*** [A]	***36.3–36.9*** [A]	***36.6*** [B]	***36.2–37.0*** [B]	***36.7*** [C]	***36.4–37.1*** [C]	≤ 0.01
Maternal fever (≥ 38.0 °C) †	12 [A]	12.4 [A]	20 [B]	11.2 [B]	1482 [C]	5.6 [C]	≤ 0.01
Thick meconium	32	28.3	65	32.0	2182	7.9	≤ 0.01
Sentinel event	6	5.3	8	3.9	130	0.5	≤ 0.01
Birth trauma	3	2.7	3	1.5	886	3.2	0.357
**Delivery mode**							
Caesarean section	113	100.0	203	100.0	798	2.9	–
Instrumental vaginal	–	–	–	–	8044	29.1	–
Spontaneous vaginal	–	–	–	–	18,769	68.0	–
**MATERNAL CHARACTERISTICS**							
**Demographics**							
Age (years)	*31*	*27–35*	*31*	*27–35*	*30*	*26–34*	0.016
≤ 18 years	3	2.7	7	3.4	931	3.4	0.913
≥ 35 years	34	30.1	56	27.5	6402	23.2	0.076
Nulliparity	82	72.6	153	75.4	14,901	54.0	≤ 0.01
Nulliparity & age ≥ 35 years	20	17.7	34	16.7	2377	8.6	≤ 0.01
Body mass index (kg/m^2)	***23.7*** [D]	***22.0–27.3*** [D]	***24.6*** [E]	***22.0–29.3*** [E]	***24.1*** [F]	***21.4–27.7*** [F]	0.110
< 18.5 kg/m^2 (underweight)	4 [D]	4.6 [D]	7 [E]	4.2 [E]	1234 [F]	5.7 [F]	0.670
18.5-24.9 kg/m^2 (normal weight)	48 [D]	55.2 [D]	78 [E]	47.3 [E]	11,180 [F]	51.3 [F]	0.451
25.0-29.9 kg/m^2 (overweight)	21 [D]	24.1 [D]	45 [E]	27.3 [E]	5515 [F]	25.3 [F]	0.820
≥ 30 .0 kg/m^2 (obese)	12 [D]	13.8 [D]	35 [E]	21.2 [E]	3615 [F]	16.6 [F]	0.220
**Antepartum risk factors**							
Preeclampsia	2	1.8	9	4.4	927	3.4	0.449
Hypertension	1	0.9	2	1.0	208	0.8	0.919
Gestational diabetes	0	0.0	1	0.5	347	1.3	0.303

Supplementary Table 1. *(Continued)*

	**Emergency caesarean section, *EmergencyCS* (*n* = 113)**		**Urgent caesarean section, *UrgentCS* (*n* = 203)**		**All *Others* (*n* = 27,611)**		
	n or *median*	% or *IQR*	n or *median*	% or *IQR*	n or *median*	% or *IQR*	p-value
**NEONATAL CHARACTERISTICS**							
Male	72	63.7	112	55.2	14,072	51.0	0.013
Gestational age (weeks)	*40*	*39–41*	*40*	*39–41*	*40*	*39–41*	≤ 0.01
36 weeks	4	3.5	4	2.0	902	3.3	0.576
Early term (37–38 weeks)	10	8.8	26	12.8	5508	19.9	≤ 0.01
Full term (39–40 weeks)	48	42.5	89	43.8	13,679	49.5	0.089
Post term (≥ 41 weeks)	51	45.1	84	41.4	7522	27.2	≤ 0.01
Birth weight (gram)	*3300*	*2991–3861*	*3375*	*2968–3760*	*3414*	*3090–3738*	0.390
Small for gestational age ‡	9	8.0	13	6.4	427	1.5	≤ 0.01
Large for gestational age ‡	7	6.2	5	2.5	1180	4.3	0.266
**DELIVERY OUTCOME**							
**Objective fetal compromise**							
Severe compromise §	5	4.4	7	3.4	155	0.6	≤ 0.01
Resuscitation	4	3.5	9	4.4	199	0.7	≤ 0.01
Apgar score < 4 at 1 min	9	8.0	24	11.8	591 [G]	2.1 [G]	≤ 0.01
Apgar score < 7 at 5 min	3	2.7	8	3.9	229 [H]	0.8 [H]	≤ 0.01
Arterial umbilical cord pH	*7.23* [I]	*7.15–7.28* [I]	*7.22* [I]	*7.12–7.27* [I]	*7.22* [J]	*7.14–7.28* [J]	≤ 0.01
pH < 7.00	6 [I]	2.8 [I]	9 [I]	4.6 [I]	190 [J]	1.0 [J]	≤ 0.01
pH < 7.05	9 [I]	8.4 [I]	23 [I]	11.7 [I]	502 [J]	2.5 [J]	≤ 0.01
**Mortality**							
Stillbirth	0	0.0	0	0.0	0	0.0	–
Neonatal death	1	0.9	0	0.0	16	0.1	≤ 0.01
**Morbidity**							
Convulsions	3	2.7	1	0.5	43	0.2	≤ 0.01
Meconium aspiration syndrome	9	8.0	7	3.4	78	0.3	≤ 0.01
NICU admission	12	10.6	32	15.8	1133	4.1 [H]	≤ 0.01
Length of stay (days)	*6*	*2–10*	*4*	*2–7*	3	*1–5*	0.109

Supplementary Table 1. *(Continued)*

	**Emergency caesarean section, *EmergencyCS* (*n* = 113)**		**Urgent caesarean section, *UrgentCS* (*n* = 203)**		**All *Others* (*n* = 27,611)**		
	n or *median*	% or *IQR*	n or *median*	% or *IQR*	n or *median*	% or *IQR*	p-value
**COMPUTERISED CTG FEATURES (FIRST HOUR)**							
Baseline (bpm)	*138* [K]	*129–148* [K]	*139* [L]	*130–148* [L]	*135* [M]	*127–142* [M]	≤ 0.01
≥ 150 bpm	25 [K]	22.1 [K]	38 [L]	18.9 [L]	2299 [M]	8.4 [M]	≤ 0.01
≥ 155 bpm	20 [K]	17.9 [K]	22 [L]	10.9 [L]	1149 [M]	4.2 [M]	≤ 0.01
≥ 160 bpm	14 [K]	12.5 [K]	11 [L]	5.5 [L]	585 [M]	2.1 [M]	≤ 0.01
Short-term variability	***5.4*** [K]	***2.9–9.4*** [K]	***4.6*** [L]	***3.1–6.9*** [L]	***6.1*** [M]	***4.5–7.9*** [M]	≤ 0.01
< 3 msec	30 [K]	26.8 [K]	44 [L]	21.9 [L]	1358 [M]	5.0 [M]	≤ 0.01
< 5 msec	50 [K]	44.6 [K]	110 [L]	54.7 [L]	8665 [M]	31.8 [M]	≤ 0.01
Long-term variability	***3.9*** [K]	***2.5–6.3*** [K]	***3.7*** [N]	***2.8–4.9*** [N]	***4.9*** [O]	***3.8–6.2*** [O]	≤ 0.01
< 3 bpm	40 [K]	35.7 [K]	60 [N]	30.0 [N]	2961 [O]	10.9 [O]	≤ 0.01
< 5 bpm	76 [K]	67.9 [K]	155 [N]	77.5 [N]	14,143 [O]	52.0 [O]	≤ 0.01
Nonreactive trace	9 [K]	8.0 [K]	20 [L]	10.0 [L]	624 [P]	2.3 [P]	≤ 0.01
Accelerations	***0*** [K]	***0–17*** [K]	***0*** [L]	***0–17*** [L]	***16*** [P]	***7–20*** [P]	≤ 0.01
≥ 1 in 15 minutes	51 [K]	45.5 [K]	96 [L]	47.8 [L]	20,572 [P]	75.5 [P]	≤ 0.01
Decelerations	***3*** [L]	***2–4*** [L]	***2*** [Q]	***1–3*** [Q]	***1*** [R]	***0–2*** [R]	≤ 0.01
≥1 in 15 minutes	103 [L]	92.8 [L]	150 [Q]	75.8 [Q]	16243 [R]	61.29 [R]	≤ 0.01
Prolonged decelerations	***0*** [K]	***0–0*** [K]	***0*** [L]	***0–0*** [L]	***0*** [P]	***0–0*** [P]	≤ 0.01
≥ 1 in 60 minutes	18 [K]	16.0 [K]	23 [L]	11.4 [L]	1388 [P]	5.1 [P]	≤ 0.01
Decelerative capacity (bpm)	***3.2*** [K]	***2.0–4.6*** [K]	***2.3*** [L]	***1.6–3.2*** [L]	***2.5*** [M]	***1.9–3.3*** [M]	≤ 0.01
< 1.0 bpm	3 [K]	2.7 [K]	11 [L]	5.5 [L]	664 [M]	2.5 [M]	≤ 0.01
< 1.5 bpm	11 [K]	9.8 [K]	39 [L]	19.4 [L]	3460 [M]	12.7 [M]	≤ 0.01
Uterine contractions	***3*** [S]	***2–3*** [S]	***2*** [T]	***2–3*** [T]	***2*** [U]	***2–3*** [U]	0.055
> 5 in 10 minutes (tachysystole)	0 [S]	0.0 [S]	0 [T]	0.0 [T]	0 [U]	0.0 [U]	–

n = number, IQR = inter-quartile range (25th - 75th percentiles), NICU = neonatal intensive care unit, bpm = beats per minute, msec = milliseconds

Super-indices *A – W* indicate the number of missing data: *A - 16, B - 24, C - 1074, D - 26, E - 38, F - 5823, G - 8, H - 11, I - 6, J - 7811, K - 1, L - 2, M - 362, N - 3, O - 388, P - 359, Q - 3, R - 752, S - 10, T - 13, U - 2340*

* Maximum maternal temperature measured during labour

† Maternal fever defined as one-time measurement of 38.0 °C, based on UK and Dutch maternity guidelines (23,24)

‡ Small for gestational age defined as birthweight < 3rd percental, large for gestational age defined as birthweight > 97th percentile, based on adjusted Yudkin's chart percentiles (26)

§ Composite outcome: stillbirth, neonatal death, seizures, neonatal encephalopathy, intubation or resuscitation followed by NICU admission for ≥ 48 h

Supplementary Table 2. Clinical and CTG characteristics of severely compromised newborns (n = 167)

	**Emergency caesarean section, *EmergencyCS* (*n* = 5)**		**Urgent caesarean section, *UrgentCS* (*n* = 7)**		**All *Others* (*n* = 155)**		
	n or *median*	% or *IQR*	n or *median*	% or *IQR*	n or *median*	% or *IQR*	p-value
**LABOUR DETAILS**							
**Labour onset**							
Induced labour	2	40.0	6	85.7	77	49.7	0.155
Established labour at start CTG	5	100.0	5	71.4	54	34.8	≤ 0.01
**Intrapartum risk factors**							
Augmentation with oxytocin	0	0.0	2	28.6	73	47.1	0.077
Maternal temperature (°C) *	*36.9*	*36.8–36.9*	*36.9* [A]	*36.2–37.1* [A]	*37.0* [B]	*36.6–37.3* [B]	0.636
Maternal fever (≥ 38.0 °C) †	0	0.0	1 [A]	16.7 [A]	13 [B]	8.6 [B]	0.618
Thick meconium	5	100.0	4	57.1	37	23.9	≤ 0.01
Sentinel event	0	0.0	0	0.0	1	0.6	0.962
Birth trauma	2	40.0	1	14.3	22	14.2	0.281
**Delivery mode**							
Caesarean section	5	100.0	7	100.0	19	12.3	–
Instrumental vaginal	–	–	–	–	62	40.0	–
Spontaneous vaginal	–	–	–	–	74	47.7	–
**MATERNAL CHARACTERISTICS**							
**Demographics**							
Age (years)	*31*	*22–37*	*34*	*30–37*	*31*	*27–35*	0.676
≤ 18 years	0	0.0	1	14.3	10	6.5	0.597
≥ 35 years	2	40.0	3	42.9	44	28.4	0.619
Nulliparity	3	60.0	6	85.7	111	71.6	0.601
Nulliparity & age ≥ 35 years	1	20.0	3	42.9	28	18.1	0.265
Body mass index (kg/m^2)	*23.8*	*22.4–25.3*	*26.9* [A]	*24.2–33.9* [A]	*24.4* [C]	*22.0–28.7* [C]	0.502
< 18.5 kg/m^2 (underweight)	0	0.0	0 [A]	0.0 [A]	6 [C]	5.2 [C]	0.742
18.5-24.9 kg/m^2 (normal weight)	4	80.0	3 [A]	50.0 [A]	57 [C]	49.1 [C]	0.401
25.0-29.9 kg/m^2 (overweight)	1	20.0	1 [A]	16.7 [A]	27 [C]	23.3 [C]	0.921
≥ 30 .0 kg/m^2 (obese)	0	0.0	2 [A]	33.3 [A]	25 [C]	21.6 [C]	0.391
**Antepartum risk factors**							
Preeclampsia	0	0.0	1	14.3	14	9.0	0.693
Hypertension	0	0.0	0	0.0	1	0.6	0.962
Gestational diabetes	0	0.0	0	0.0	3	1.9	0.889

Supplementary Table 2. *(Continued)*

	**Emergency caesarean section, *EmergencyCS* (*n* = 5)**		**Urgent caesarean section, *UrgentCS* (*n* = 7)**		**All *Others* (*n* = 155)**		
	n or *median*	% or *IQR*	n or *median*	% or *IQR*	n or *median*	% or *IQR*	p-value
**NEONATAL CHARACTERISTICS**							
Male	2	40.0	4	57.1	87	56.1	0.772
Gestational age (weeks)	*39*	*38–40*	*41*	*40–41*	*39*	*38–41*	0.184
36 weeks	0	0.0	0	0.0	9	5.8	0.692
Early term (37–38 weeks)	2	40.0	1	14.3	35	22.6	0.567
Full term (39–40 weeks)	2	40.0	1	14.3	64	41.3	0.362
Post term (≥ 41 weeks)	1	20.0	5	71.4	47	30.2	0.062
Birth weight (gram)	*4103*	*2601–4327*	*3530*	*3029–3848*	*3313*	*3032–3699*	0.673
Small for gestational age ‡	1	20.0	1	14.3	7	4.5	0.182
Large for gestational age ‡	2	40.0	0	0.0	10	6.5	0.013
**DELIVERY OUTCOME**							
**Objective fetal compromise**							
Severe compromise §	5	100.0	7	100.0	155	100.0	–
Resuscitation	3	60.0	6	85.7	98	63.2	0.470
Apgar score < 4 at 1 min	1	20.0	6	85.7	85 [A]	55.2 [A]	0.076
Apgar score < 7 at 5 min	1	20.0	4	57.1	75 [D]	49.0 [D]	0.396
Arterial umbilical cord pH	*7.14*	*7.09–7.28*	*6.83* [A]	*6.78–7.01* [A]	*7.14* [E]	*7.05–7.23* [E]	≤ 0.01
pH < 7.00	0	0.0	4 [A]	66.7 [A]	26 [E]	19.3 [E]	≤ 0.01
pH < 7.05	1	20.0	5 [A]	88.3 [A]	33 [E]	24.4 [E]	≤ 0.01
**Mortality**							
Stillbirth	0	0.0	0	0.0	0	0.0	–
Neonatal death	1	20.0	0	0.0	14	9.0	0.488
**Morbidity**							
Convulsions	3	60.0	1	14.3	43	27.7	0.203
Meconium aspiration syndrome	5	100.0	1	14.3	19	12.3	≤ 0.01
NICU admission	5	100.0	6	85.7	131	84.5	0.633
Length of stay (days)	*9*	*8–18*	*5*	*4–6*	***3***	*3–8*	0.168

Supplementary Table 2. *(Continued)*

	**Emergency caesarean section, *EmergencyCS* (*n* = 5)**		**Urgent caesarean section, *UrgentCS* (*n* = 7)**		**All *Others* (*n* = 155)**		
	n or *median*	% or *IQR*	n or *median*	% or *IQR*	n or *median*	% or *IQR*	p-value
**COMPUTERISED CTG FEATURES (FIRST HOUR)**							
Baseline (bpm)	*158*	*145–164*	*135*	*127–145*	*136* [D]	*129–144* [D]	0.014
≥ 150 bpm	3	60.0	1	14.3	21 [D]	13.7 [D]	0.027
≥ 155 bpm	3	60.0	0	0.0	16 [D]	10.5 [D]	≤ 0.01
≥ 160 bpm	2	40.0	0	0.0	8 [D]	5.2 [D]	≤ 0.01
Short-term variability	*1.8*	*1.6–2.7*	5.6	*3.2–7.0*	*5.3* [D]	*4.1–7.8* [D]	≤ 0.01
< 3 msec	4	80.0	1	14.3	14 [D]	9.2 [D]	≤ 0.01
< 5 msec	5	100.0	3	42.9	61 [D]	39.9 [D]	0.047
Long-term variability	*2.1*	*1.6–2.6*	*4.4*	*2.1–5.2*	*4.6* [D]	*3.4–6.1* [D]	≤ 0.01
< 3 bpm	5	100.0	3	42.9	25 [D]	16.3 [D]	≤ 0.01
< 5 bpm	5	100.0	5	71.4	83 [D]	54.3 [D]	0.091
Nonreactive trace	2	40.0	2	28.6	9 [D]	5.9 [D]	≤ 0.01
Accelerations	*0*	*0–0*	*0*	*0–15*	*16* [D]	*0–19* [D]	0.013
≥ 1 in 15 minutes	0	0.0	3	42.9	102 [D]	66.7 [D]	0.005
Decelerations	*2*	*1–3*	*1*	*0–2*	*1* [A]	*0–2* [A]	0.125
≥ 1 in 15 minutes	5	100.0	5	71.4	80 [F]	52.6 [F]	0.075
Prolonged decelerations	*0*	*0–2*	*0*	*0–0*	*0* [D]	*0–0* [D]	0.012
≥ 1 in 60 minutes	2	40.0	1	14.3	9 [D]	5.8 [D]	0.012
Decelerative capacity (bpm)	*1.8*	*1.0–2.4*	*2.3*	*1.2–3.8*	*2.4* [D]	*1.7–3.1* [D]	0.301
< 1.0 bpm	1	20.0	2	28.6	3 [D]	2.0 [D]	≤ 0.01
< 1.5 bpm	2	40.0	2	28.6	26 [D]	17.0 [D]	0.324
Uterine contractions	*2*	*1–3*	*3*	*1–3*	*2*	*2–3*	0.621
> 5 in 10 minutes (tachysystole)	0	0.0	0	0.0	0	0.0	–

Supplementary Table 2. *(Continued)*

	Emergency caesarean section, *EmergencyCS* (*n* = 5)		Urgent caesarean section, *UrgentCS* (*n* = 7)		All *Others* (*n* = 8/155) ◊		
	n or *median*	% or *IQR*	n or *median*	% or *IQR*	n or *median*	% or *IQR*	p-value
**VISUAL CTG EVALUATION (FIRST HOUR)**							
**2015 FIGO classification system**							
Normal	0	0.0%	4	57.1%	4	50.0%	0.104
Suspicious	1	20.0%	0	0.0%	0	0.0%	0.206
Pathological	4	80.0%	3	42.9%	4	50.0%	0.415
**Abnormalities not detectable with IA**	0	0.0%	2 [F]	66.7% [F]	2 [B]	50.0% [B]	0.211

n = number, IQR = inter-quartile range (25th - 75th percentiles), NICU = neonatal intensive care unit, bpm = beats per minute, msec = milliseconds, IA = intermittent auscultation

Super-indices *A – F* indicate the number of missing data: *A - 1, B - 4*, *C - 39*, *D - 2, E - 20, F - 3*

* Maximum maternal temperature measured during labour

† Maternal fever defined as one-time measurement of 38.0 °C, based on UK and Dutch maternity guidelines (23,24)

‡ Small for gestational age defined as birthweight < 3rd percental, large for gestational age defined as birthweight > 97th percentile, based on adjusted Yudkin's chart percentiles (26)

§ Composite outcome: stillbirth, neonatal death, seizures, neonatal encephalopathy, intubation or resuscitation followed by NICU admission for ≥ 48 h

◊ 8 randomly selected first-hour traces

Supplementary Table 3. Visual CTG classification (FIGO 2015) and possibility to detect suspicious or pathological traces with intermittent auscultation (Parts et al. 2018). Performed by A.A.K. Lovers, final year medical student, Vrije Universiteit, Amsterdam, The Netherlands.

	Baseline	Variability	Decelerations	Interpretation	Detectable with intermittent auscultation
EmergencyCS					
Trace 1	Normal	Reduced	Repetitive	Pathological	No (reduced variability)
Trace 2	Tachycardia *	Reduced	Absent	Pathological	No (reduced variability)
Trace 3	Normal	Normal	Present	Suspicious	Yes (prolonged deceleration)
Trace 4	Tachycardia *	Reduced	Present	Pathological	No (reduced variability)
Trace 5	Tachycardia *	Reduced	Present	Pathological	No (reduced variability)
UrgentCS					
Trace 1	Normal	Normal	Absent	Normal	N/A
Trace 2	Normal	Reduced	Absent	Pathological	No (reduced variability)
Trace 3	Normal	Normal	Repetitive	Pathological	Yes (repetitive decelerations)
Trace 4	Normal	Normal	Normal	Normal	N/A
Trace 5	Normal	Normal	Absent	Normal	N/A
Trace 6	Normal	Normal	Absent	Normal	N/A
Trace 7	Tachycardia *	Reduced	Absent	Pathological	No (reduced variability)
Others					
Trace 1	Normal	Normal	Absent	Normal	N/A
Trace 2	Normal	Reduced	Absent	Pathological	No (reduced variability)
Trace 3	Normal	Normal	Absent	Normal	N/A
Trace 4	Bradycardia	Normal	Absent	Pathological	Yes (bradycardia)
Trace 5	Tachycardia *	Reduced	Absent	Pathological	No (reduced variability)
Trace 6	Tachycardia *	Normal	Absent	Normal	N/A
Trace 7	Tachycardia *	Normal	Absent	Normal	N/A
Trace 8	Normal	Increased	Repetitive	Pathological	Yes (repetitive decelerations)

EmergencyCS - emergency caesarean delivery (< 2 hours after start CTG monitoring), *UrgentCS* - unplanned caesarean delivery (between 2 to 5 hours after start CTG monitoring), N/A - not applicable, * baseline ≥ 150 bpm
